# Endogenous ROS production in early differentiation state suppresses endoderm differentiation via transient FOXC1 expression

**DOI:** 10.1038/s41420-022-00961-2

**Published:** 2022-04-01

**Authors:** Sugako Oka, Teruhisa Tsuzuki, Masumi Hidaka, Mizuki Ohno, Yoshimichi Nakatsu, Mutsuo Sekiguchi

**Affiliations:** 1 Frontier Research Center, Fukuoka Dental College, Fukuoka, 814-0193 Japan; 2 Department of Medical Biophysics and Radiation Biology, Faculty of Medical Science, Kyushu University, 3-1-1, Maidashi, Higashiku, Fukuoka, 812-8582 Japan; 3 Department of Physiological Science and Molecular Biology, Fukuoka Dental College, Fukuoka, 819-0193 Japan; 4 Oral Medicine Research Center, Fukuoka Dental College, Fukuoka, 819-0193 Japan

**Keywords:** Stem-cell differentiation, Cancer stem cells

## Abstract

Oxidative stress plays a pivotal role in the differentiation and proliferation of cells and programmed cell death. However, studies on the role of oxidative stress in differentiation have mainly employed the detection of reactive oxygen species (ROS) during differentiation or generated by ROS inducers. Therefore, it is difficult to clarify the significance of endogenous ROS production in the differentiation of human cells. We developed a system to control the intracellular level of ROS in the initial stage of differentiation in human iPS cells. By introducing a specific substitution (I69E) into the SDHC protein, a component of the mitochondrial respiratory chain complex, the endogenous ROS level increased. This caused impaired endoderm differentiation of iPS cells, and this impairment was reversed by overproduction of mitochondrial-targeted catalase, an anti-oxidant enzyme. Expression of tumor-related FOXC1 transcription factor increased transiently as early as 4 h after ROS-overproduction in the initial stage of differentiation. Knockdown of FOXC1 markedly improved impaired endoderm differentiation, suggesting that endogenous ROS production in the early differentiation state suppresses endoderm differentiation via transient FOXC1 expression.

## Introduction

Reactive oxygen species (ROS) are generated by utilizing oxygen for ATP production during cellular respiration. The electrons released from the mitochondrial respiratory chain react with surrounding oxygen molecules to yield superoxide (O_2_^−^), which can be converted to hydrogen peroxide (H_2_O_2_). H_2_O_2_ molecules are transformed to hydroxyl radicals (•OH) by the Fenton reaction. An anti-oxidant enzyme, catalase, converts H_2_O_2_ to water, resulting in the suppression of endogenous ROS production. ROS may play pivotal roles in the differentiation and proliferation of cells. In mouse and human pluripotent stem cells, although levels of ROS remain low to maintain stemness [1, 2], elevated ROS production occurs during differentiation. ROS regulate the differentiation of mesenchymal stem cells into many types of cells, including adipocytes, chondrocytes, osteocytes, and neuronal cells, and further control the differentiation of ES cells into cardiomyocytes [3, 4]. Various signals including transcriptional factors such as p53 and HIF are involved in ROS-induced differentiation into the three germ layers [5–9]. In contrast, it was reported that excess ROS production due to a ROS inducer blocked stem cell differentiation, including mesenchymal stromal cells, via the suppression of osteogenic signal pathways [3]. From these findings, the threshold of ROS levels to induce stem cell differentiation might be tightly regulated. ROS increase during aging by mitochondrial dysfunction or reduced anti-oxidant enzyme activity, and may cause age-related disease including cancer or neurodegenerative disorders; therefore, it is important to clarify the role of excess ROS production in stem cell differentiation. However, it is difficult to time-specifically control endogenous ROS production within human stem cells, and studies of the role of ROS in differentiation have mainly employed ROS blockers/activators as chemical agents.

**Fig. 1:**
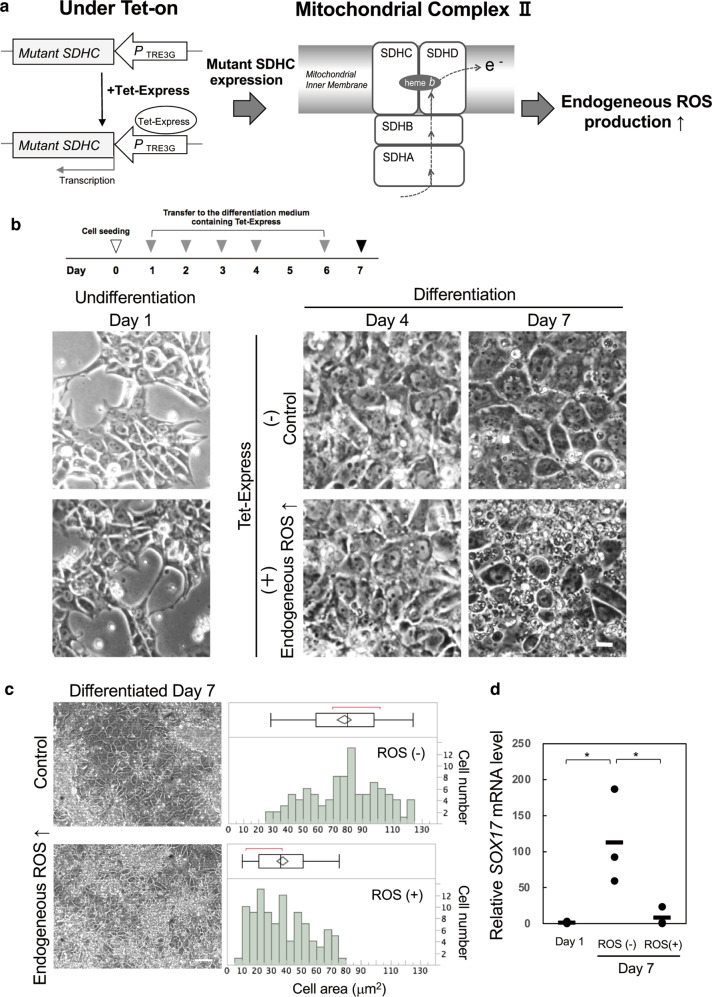
Impaired differentiation by endogenous ROS production in early endoderm differentiation state. **a** To induce ROS overproduction, mutant I69E SDHC protein was expressed under the Tet-on (Tet-express) system. Mitochondrial respiratory chain complex II manipulation by mutant SDHC released a large number of electrons during respiration that reacted with surrounding oxygen molecules to yield superoxide (O_2_^−^), which can be converted to hydrogen peroxide (H_2_O_2_), resulting in endogenous ROS production. **b** Endoderm differentiation in human iPS cells producing ROS in the early differentiation state. ChiPSC12-M cells were cultured for 7 days in endoderm differentiation medium to differentiate into definitive endoderm. The medium was exchanged to the fresh one on Day 1, 2, 3, 4, and 6. To express mutant-form SDHC, cells were cultured in the differentiation medium containing Tet-Express solution on Day 1 as an early differentiation state. From Days 1 to 7, the cells were cultured with the differentiation medium with or without Tet-Express solution. Left panels on Day 1 show the undifferentiated ChiPSC12-M cells pre-replacement of the differentiation medium. Upper panels: cells cultured with the medium without Tet-Express solution exhibited normal definitive endoderm differentiation on Day 7. Lower panels: cells cultured with the medium with Tet-Express solution from Days 2 to 7 exhibited markedly impaired differentiation. Scale bar, 20 μm. Results from one of four independent experiments are presented. **c** Day 7 of differentiation in ChiPSC12-M cells. Cells were cultured in the differentiation medium with or without Tet-Express solution for 7 days. Scale bar, 100 μm. The cell area of 100 cells in the left panels was measured and is shown in the histogram and outlier box plot. Results from one of three independent experiments are presented. **d** Expression level of an endoderm marker, *SOX17* mRNA, by real-time RT-PCR analysis. ChiPSC12-M cells were cultured with or without Tet-Express solution (ROS [+] or ROS [−]) for 7 days. Day 1 shows the undifferentiated cells pre-replacement of the differentiation medium. Bars represent the means. *N* = 3. Wilcoxon exact test (two-sided), *p = 0.0286.

Recently, we reported the establishment of a human iPS cell line producing ROS by manipulating the SDHC subunit of mitochondrial respiratory chain complex II under the Tet-on system (ChiPSC12-M cells, Fig. 1a) [10]. Respiratory chain complex II is composed of four protein subunits (SDHA, SDHB, SDHC, and SDHD). In the mouse, V69E mutation of the *Sdhc* gene encoding mitochondrial succinate-ubiquinone oxidoreductase caused neonatal growth retardation by mitochondrial oxidative stress [11, 12]. We established ChiPSC12-M cells overproducing corresponding human mutant I69E SDHC protein by the advanced Tet-on system with the transcriptional activator protein Tet-Express (Takara Bio Inc.) and confirmed ROS production in a Tet-Express-dose-dependent manner. In the present study, we examined the effect of ROS overproduction on differentiation using ChiPSC12-M cells. Furthermore, we developed human iPS cells exhibiting a change in the intracellular ROS level by manipulating both the ROS-yielding mutant SDHC and ROS-degenerating catalase production (CHiPSC12-CAT cells). These human iPS cell lines may be useful for clarifying the role of endogenous ROS overproduction in the early stage of differentiation and molecular pathways leading to age-related disease.

## Results

### Overproduction of endogenous ROS causes impaired definitive endoderm differentiation

We first examined whether excess endogenous ROS in the early stage of differentiation affects the development of human iPS cells. ChiPSC12 cells can differentiate towards definitive endoderm via extraembryonic endoderm for 7 days by culturing them in endoderm differentiation medium. ChiPSC12-M cells were plated at a seeding density of 2 × 10^5^ cells/cm^2^ in 24-well plate. ChiPSC12-M cells were cultured for 7 days by changing the differentiation medium with or without Tet-Express, mutant SDHC inducer on Days 1, 2, 3, 4, and 6, according to the manufacturer’s instructions. The cell plate on each differentiation day was washed twice with differentiation medium, and then images were acquired and analyzed. On Day 7, ChiPSC12-M cells without Tet-Express were able to differentiate into definitive endoderm, showing the arranged trapezoidal cell morphology with a large cell body (Fig. 1b, Day 7, Tet-Express [−]). In contrast, cells subjected to Tet-Express treatment exhibited impaired differentiation with a small and irregularly aligned cell morphology (Fig. 1b, Day 7, Tet-Express [+]). As shown on Day 7 in Fig. 1c, histogram analysis of each cell area (the area occupied by a single cell) indicated that cells overproducing ROS showed a decrease in the cell area (~80 μm^2^ in the lower graph) as an effect of impaired differentiation compared with untreated cells (~125 μm^2^ in the upper graph). On differentiation Day 7, real-time RT-PCR analysis revealed that the expression level of an endoderm marker, *SOX17*, was increased in cells without Tet-Express (Fig. 1d, ROS [−]). On the other hand, overproducing ROS in the early differentiation state markedly reduced *SOX17* expression, confirming the impaired development (Fig. 1d ROS [+]). These data suggest that excess endogenous ROS in the initial stage of differentiation impairs the efficiency of differentiation towards definitive endoderm.

### Establishment of human iPS cells exhibiting a reversible change in endogenous ROS production

**Fig. 2:**
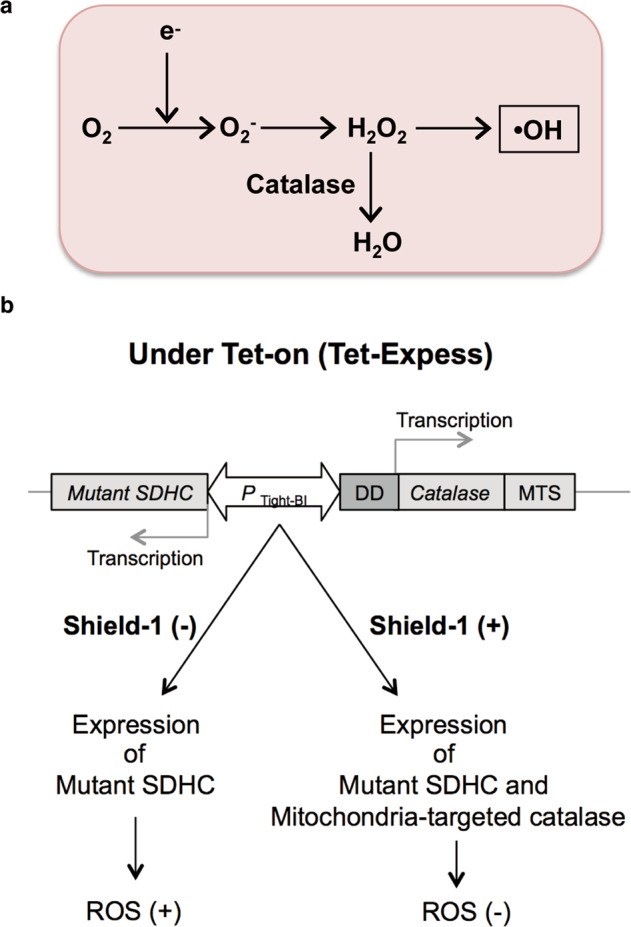
Construction of human iPS cells exhibiting a change in endogenous ROS production. **a** The electrons released from the respiratory chain react with surrounding oxygen molecules to yield superoxide (O_2_^−^), which can be converted to hydrogen peroxide (H_2_O_2_), and H_2_O_2_ molecules are transformed to hydroxyl radicals (•OH) by the Fenton reaction. Catalases convert H_2_O_2_ to water, resulting in the suppression of ROS production. **b** Construction of human iPS cells exhibiting a change in endogenous ROS production due to regulation of the expression of mutant SDHC or both mutant SDHC and mitochondrial-targeted human catalase under the Tet-ProteoTuner system. In the presence of Tet-Express, both SDHC (I69E) and DD (destabilization domain)-Tag fusion mt-catalase are expressed. In the absence of a small membrane-permeable ligand, Shield-1 protein, DD-Tag fusion mt-catalase is degraded by proteasomes, resulting in ROS overproduction. Shield-1 binds to DD-Tag and protects mt-catalase from proteasomal degradation, whereas both SDHC (I69E) and mt-catalase are stably expressed in identical cells, resulting in ROS inhibition. MTS: mitochondrial targeting sequence. P_tight_-BI: Tet-responsive P_tight_ promoter, which contains a modified Tet response element.

**Fig. 3:**
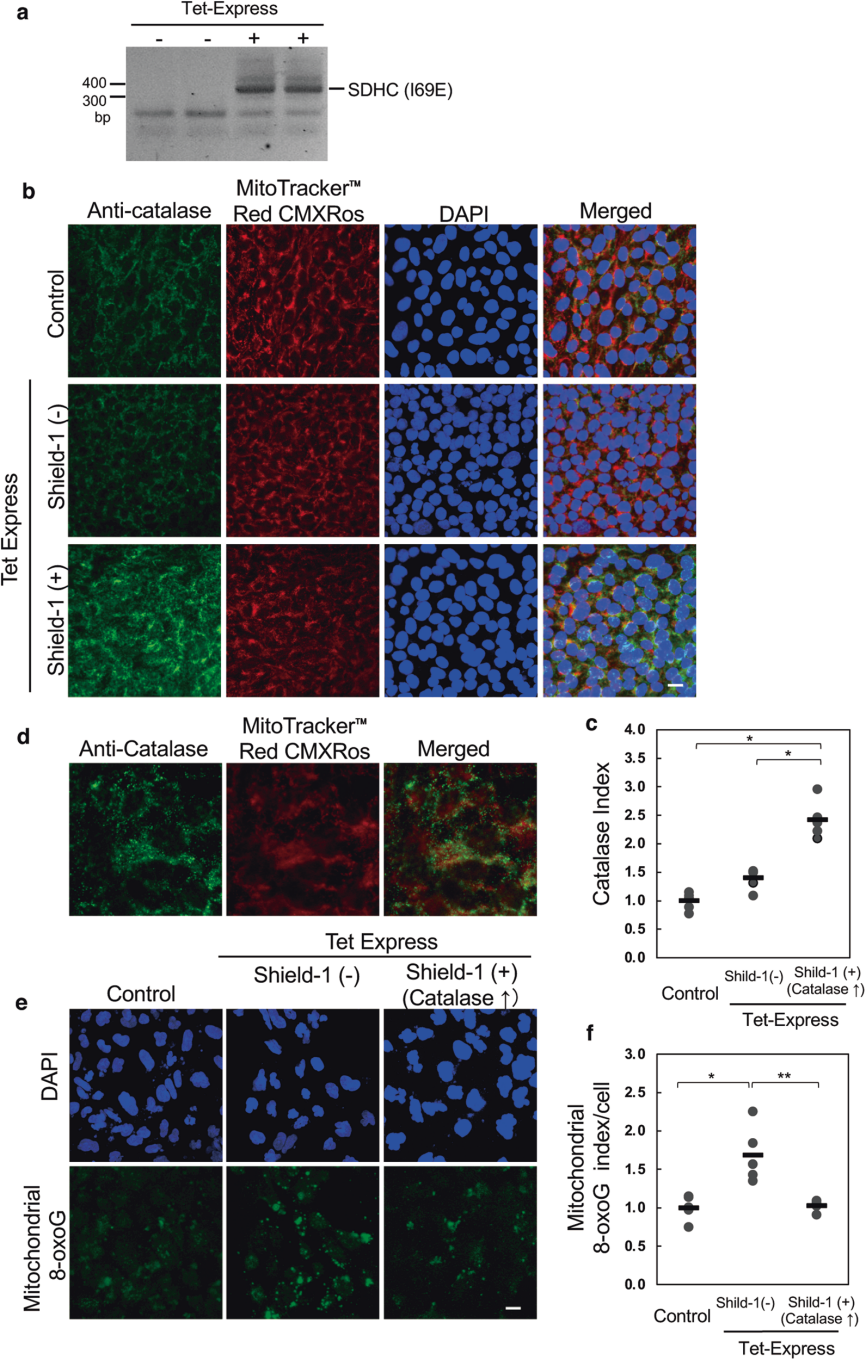
Establishment of human iPS cells exhibiting a change in endogenous ROS production. **a** The expression of I69E mutant SDHC transcripts. pTRE-Cycle1 that encodes both SDHC (I69E) and DD-Tagged mitochondrial-targeted human catalase was introduced into the ChiPSC12 cells, and stable transfectants (ChiPSC12-CAT cells) were selected. ChiPSC12-CAT cells express I69E mutant SDHC under treatment with an SDHC inducer (Tet-express). To discriminate the I69E mutant transcripts, cDNA from each of the duplicate cell cultures was amplified using mutant SDHC-specific primer sets. **b** Expression of human catalase was confirmed by immunostaining using anti-catalase antibody in ChiPSC12-CAT cells. Cells were cultured with 400 μL of the medium containing 10 μL of Tet-Express solution for 24 h and treated with 0.8 μL of Shield-1 protein for 3.5 h. Control: cells cultured without Tet-Express or Shield-1. Shield-1 (−): cells cultured with only Tet-Express. Shield-1 (+): cells cultured with both Tet-Express and Shield-1. MitoTracker TM red CXMRos or DAPI is a mitochondrial or nuclear marker. Scale bar, 10 μm. **c** Increased human catalase expression in ChiPSC12-CAT cells by Shield-1 protein treatment. Fluorescence intensities of catalase in the five digital images of each cell were measured, and the relative catalase index is shown as a dot plot. Bars represent the means. One-way ANOVA: *p* < 0.0001. Tukey–Kramer HSD test: *p < 0.0001. **d** Expression of human catalase in the mitochondria of ChiPSC12-CAT cells. Magnified image of ChiPSC12-CAT cells after treatment with Tet-Express and Scield-1. In the merged image, the yellow signal indicates the expression of mitochondrial-targeted human catalase. **e** Overexpression of mitochondrial catalase markedly suppressed the accumulation of mitochondrial 8-oxoG in ChiPSC12-CAT cells under Tet-Express treatment. Cells were cultured in differentiation media containing Tet-Express solution with or without Shield-1 treatment for 7 days. Control: cells cultured with differentiation medium without Tet-Express or Shield-1. To solely detect 8-oxoG in mitochondrial DNA, cultured slides were pretreated with 5 mg/mL RNase and subjected to immunostaining with anti-8-oxo-dG antibody. Scale bar, 10 μm. **f** Measurement of 8-oxoG in cells. Fluorescence intensities of 8-oxoG in the five digital images of each treated cell in Fig. 3e were measured. The mitochondrial 8-oxoG was calculated by subtracting the nuclear 8-oxoG immunofluorescence from immunofluorescence of the whole cell and the mitochondrial 8-oxoG index is shown as a dot plot. Bars represent the means. One-way ANOVA: *p* = 0.0009. Tukey–Kramer HSD test: *p = 0.0016, **p = 0.0025.

To assess whether the impaired differentiation was induced by an elevated level of endogenous ROS, we next examined whether overexpression of human catalase could rescue the impaired differentiation. Human catalase, an anti-oxidant enzyme, is typically located in peroxisomes and converts hydrogen peroxide (H_2_O_2_) to H_2_O, resulting in the inhibition of ROS accumulation (Fig. 2a). Since mitochondria are the main source of ROS generation, overexpression of human catalase in mitochondria protects HepG2 cells against oxidative damage and extends the mouse lifespan, accompanied by the attenuation of ROS production [13, 14]. We established human iPS cell lines expressing only mutant SDHC (I69E) protein or both mutant SDHC (I69E) and mitochondrial-targeted human catalase (mt-catalase) under the Tet-On ProteoTuner system (ChiPSC12-CAT cells). As shown in Fig. 2b, under treatment with Tet-Express, mutant SDHC (I69E) protein is expressed. In the absence of the small membrane-permeable ligand Shield-1 protein, mt-catalase fused with DD (destabilization domain)-Tag is degraded by proteasomes, maintaining an increase of ROS production (ROS [+]). On the other hand, Shield-1 protein binds to DD-Tag and can protect mt-catalase from proteasomal degradation and then both SDHC (I69E) and DD-Tag fusion mt-catalase are stably expressed in the same cell, canceling the effect of ROS production (ROS [−]). Expression of the mutant *SDHC* gene was confirmed by mutant-specific RT-PCR in ChiPSC12-CAT cells in the presence of Tet-Express (Fig. 3a). In immunofluorescence staining of Fig. 3b, ChiPSC12-CAT cells treated with only Tet-Express (middle panels, Shield-1 [−]) exhibited a similar signal intensity of anti-human catalase compared with no treatment (control, upper panels). In contrast, cells treated with both Tet-Express and Shield-1 showed a marked increase of the signal intensity (lower panels, Shield-1 [+]). The dot plot of the relative catalase index revealed catalase overexpression in ChiPSC12-CAT cells by Shield-1 protein treatment (Fig. 3c). As shown in the magnified image of Fig. 3d, the green anti-catalase signal was partially co-localized with Mito Tracker Red CMXros, a mitochondrial marker, which confirmed the stabilization of mt-catalase protein by Shield-1 treatment in mitochondria. In the merged image, the expression of mt-catalase was observed as a yellow signal, and the authentic catalase in peroxisomes was shown as remaining green signals. Subsequently, we examined whether endogenous ROS production was reduced by mt-catalase overexpression (Fig. 3e). We measured the level of 8-oxoG, an oxidized form of guanine, as a ROS product by anti-8-oxo-dG immunostaining. On comparing with ChiPSC12-CAT cells without any treatment (control), the cells treated with only Tet-Express showed the accumulation of mitochondrial 8-oxoG as a perinuclear dot-like signal (middle panel, Shield-1 [−]). The accumulation was markedly suppressed by the expression of mt-catalase (right panel, shield-1 [+]). The graph in Fig. 3f shows the inhibition of 8-oxoG accumulation by mt-catalase expression. Based on these results, we could successfully establish human iPS lines exhibiting altered levels of endogenous ROS production.

### Overexpression of mitochondrial-targeted human catalase the improved impaired endoderm differentiation

**Fig. 4:**
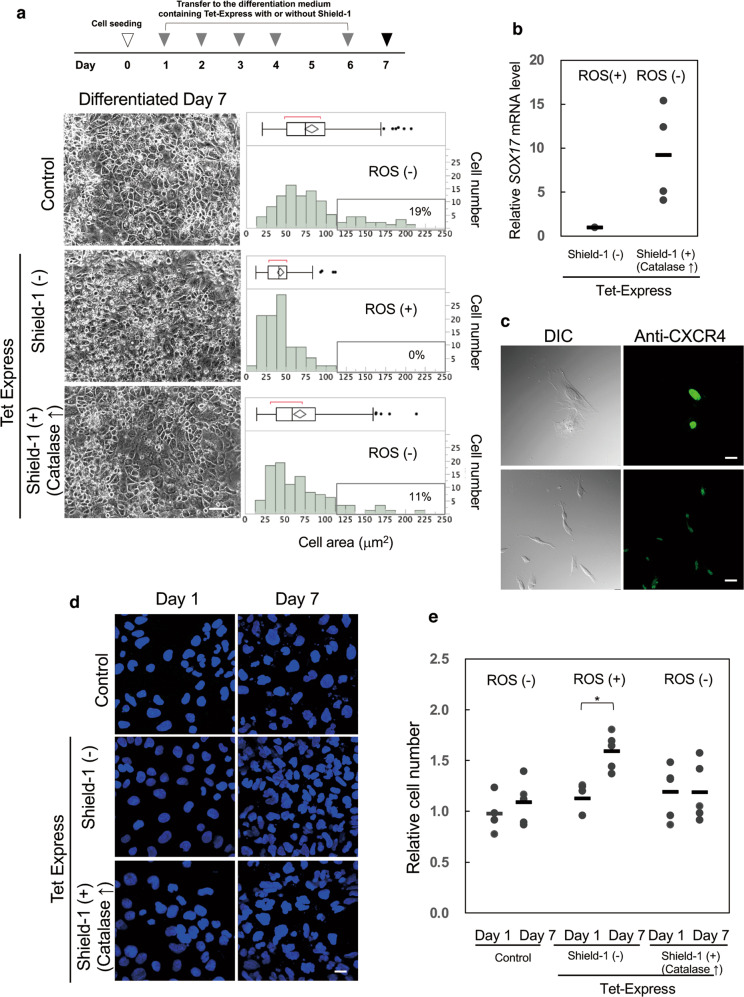
Overexpression of mitochondrial catalase improved impaired differentiation by internal ROS production. **a** Day 7 of differentiation in ChiPSC12-CAT cells. Cells were cultured with differentiation medium containing only Tet-Express or both Tet-Express and Shield-1 for 7 days. Control: cells cultured without Tet-Express or Shield-1. Shield-1 (−): cells cultured with only Tet-Express. Shield-1 (+): cells cultured with both Tet-Express and Shield-1. Scale bar, 100 μm. The cell area of 100 cells in the left panels was measured and is shown in the histogram and outlier box plot. Results from one of three independent experiments are presented. **b** Mt-catalase rescued a reduced endoderm marker, *SOX17* mRNA expression, on Day 7 of differentiation in ChiPSC12-CAT cells. Bars represent the means. *N* = 3. Wilcoxon exact test (two-sided), *p = 0.0286. **c** Immunostaining using anti-CXCR4, another endoderm marker, in control cells on Day 7. Confirmation of a positive correlation between the cell area and differentiation levels. Scale bar, 20 μm. **d** ChiPSC12-CAT cells producing ROS on treatment with Tet-Express showed cell proliferation on Day 7 of differentiation. Staining with DAPI of fixed ChiPSC12-CAT cells on Days 1 and 7. Scale bar, 10 μm. **e** Relative cell numbers on Days 1 and 7. Cell numbers in the five digital images of each treated cell in Fig. 4d were measured and relative cell number is shown as a dot plot. Bars represent the means. Wilcoxon exact test (two-sided), *p = 0.0357. Scale bar, 20 μm.

We subsequently examined the effect of mt-catalase overexpression on the impaired differentiation in human iPS cells producing ROS. As shown in Fig. 4a, on Day 7 of differentiation, cells producing ROS (middle panel, Shield-1 [−]) exhibited a decrease in the cell area (~115 μm^2^) compared with untreated control cells (upper panel), confirming the poor development caused by endogenous ROS overproduction in the early stage of differentiation. Overexpression of mt-catalase induced by Shield-1 markedly improved impaired differentiation to a level similar to that in the control (lower panel, Shield-1 [−]). Most cells producing ROS showed a small and irregularly aligned morphology and the cell area decreased to less than 115 μm^2^. On the contrary, in the control cells or cells with both Tet-Express and Shield-1 treatment, 19 or 11% of the counted cells exhibited an increased cell area of more than 115 μm^2^, respectively. The decline in the expression level of *SOX17* as an endoderm marker was rescued by catalase overexpression (Fig. 4b). As can be noted in Fig.1d, the relative expression level of SOX17 of (ROS (−)) showed an increase 13.3 times higher than that of (ROS (+)), and the expression of catalase improved this level to 67% (ROS (−)), shown in Fig. 4b. Immunofluorescence staining of control cells on Day 7 revealed that cells with a large cell body exhibited an elevated CXCR4 signal, another endoderm marker (Fig. 4c, upper panels), compared with cells with a small cell body (lower panels), confirming that the cell area on Day 7 reflects differentiation levels. To clarify whether endogenous ROS in the early stage of differentiation promotes proliferation in the absence of normal differentiation, we analyzed the number of DAPI-stained cells on Day 7 relative to that on Day 1. As shown in Fig. 4d and the graph of Fig. 4e, the relative cell number on Day 7 was markedly increased by ROS overproduction and the expression of catalase in mitochondria canceled the proliferation. These findings suggest that mitochondrial-targeted catalase improved the impaired differentiation ability via the inhibition of ROS overproduction.

### Transient expression of FOXC1 transcription factor is involved in impaired differentiation induced by ROS overproduction

**Fig. 5:**
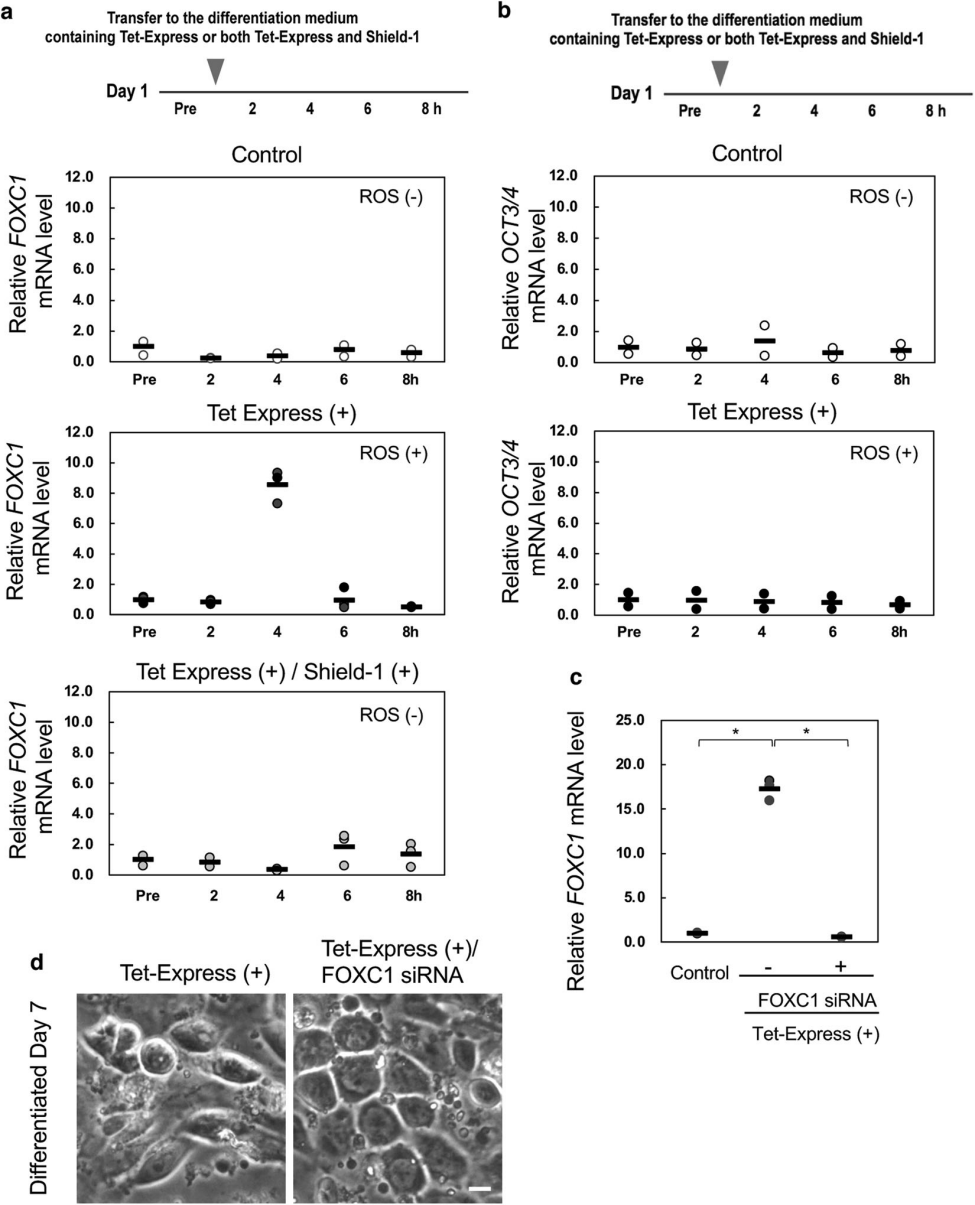
The expression of *FOXC1* was transiently increased on early differentiation in a human iPS cell line showing increased endogenous ROS production. **a** Expression level of *FOXC1* mRNA by real-time RT-PCR analysis in ChiPSC12-CAT cells. Cells were cultured for 2, 4, 6, and 8 h after replacement with Day 1 differentiation medium containing only Tet-Express (Catalase [−]) or both Tet-Express and Shield-1 (Catalase [+]). Control: cells cultured without Tet-Express or Shield-1. Bars represent the means. *N* = 3. **b** Expression level of *Oct3/4* mRNA by real-time RT-PCR analysis in ChiPSC12-CAT cells. *N* = 2. **c** Confirmation of *FOXC1* knockdown in the early stage of differentiation on Day 1 by real-time RT-PCR analysis. Control: cells cultured without Tet-Express. Cells were treated FOXC1 siRNA on Day 0 and then cultured with differentiation medium containing Tet-Express. Bars represent the means. *N* = 3. Wilcoxon exact test (two-sided), *p = 0.0286. **d** *FOXC1* knockdown in the early stage of differentiation rescued differentiation impairment on Day 7 in ChiPSC12-CAT cells producing ROS. Tet-Express: Cells were cultured with differentiation medium containing Tet-Express for 7 days. Tet-Express/*FOXC1* siRNA: cells were treated FOXC1 siRNA on Day 0 and then cultured with differentiation medium containing Tet-Express for 7 days. Scale bar, 20 μm. Results from one of three independent experiment are presented.

To clarify how endogenous ROS generation in the initial stage of differentiation inhibits definitive endoderm differentiation, we examined the alteration of gene expression on Day 1 of differentiation. ChiPSC12-CAT cells after starting differentiation were placed in Day 1 differentiation medium containing Tet-Express solution or both Tet-Express and Shield-1, cultured for 2, 4, 6, or 8 h, and then subjected to real-time RT-PCR analysis (Fig. 5a). Forkhead box C1 (FOXC1), a member of the forkhead family of transcriptional factors, is generally considered to be required for normal development, including the heart, blood vessels, kidney and urinary tract, eyes, and bones [15]. In addition, FOXC1 has a role in the maintenance of hematopoietic stem and progenitor cells in bone marrow [16] and promotes cell proliferation and invasion in human cancer [17, 18]. In ChiPSC12-CAT cells producing ROS, expression of *FOXC1* was transiently increased 4 h after Tet-Express treatment (middle panel, ROS [+]). Mt-catalase canceled the transient *FOXC1* expression (lower panel, ROS [−]). In contrast, the expression level of another transcriptional factor, *Oct 3/4*, was not increased under ROS overproduction (Fig. 5b). By *FOXC1* knockdown using siRNA, transient *FOXC1* expression 4 h after Tet-Express treatment was suppressed on Day 1 (Fig. 5c) and impaired endoderm differentiation on Day 7 was markedly improved (Fig. 5d). These results suggest that endogenous ROS production in early differentiation inhibited endoderm differentiation via transient FOXC1 expression.

## Discussion

By manipulating both the ROS-yielding mutant SDHC and ROS-degenerating catalase, we established human iPS cell lines exhibiting a change in the amount of ROS production in identical cells (CHiPSC12-CAT cells). Using this system, mitochondrial ROS production in the early stage of differentiation markedly inhibited endoderm differentiation of human iPS cells. Since originally ChiPSC 12 cell lines can be differentiated into various types of cells including hepatocytes, pancreatic beta cells, cardiomyocytes, and neural progenitors [19–22], we are able to elucidate the role of ROS in differentiation by modulating ROS production at any time-point during development.

In the present study, expression of the transcription factor FOXC1 containing a characteristic DNA-binding forkhead domain was transiently increased by ROS production in the initial stage of differentiation, and mitochondrial-targeted catalase prevented its elevation. Moreover, transient expression of FOXC1 in the early stage of differentiation is involved in suppressing endoderm differentiation. FOXC1 is commonly expressed in early paraxial mesoderm and required for normal development, including that of the heart, kidneys, and brain. FOXC1 null mice exhibited early postnatal death with hydrocephalus, eye defects, and multiple skeletal abnormalities [23–25]. It has been suggested that induction of one germ layer marker represses another germ layer to regulate cell fate. Several ectoderm markers, including the T-box transcription factor Tbx2, have been implicated in mesendodermal differentiation suppression. Xema/Foxi1e, the Foxi-subclass of winged-helix transcription factors expressed in the ectoderm, is sufficient for the inhibition of mesendoderm formation [26, 27].

Furthermore, it has been reported that the expression of *FOXC1* is increased in various types of cancer, including breast cancer, hepatocellular carcinoma, pancreatic and non-small cell lung cancers, and colon cancer and associated with cancer progression [28–30]. FOXC1 overexpression was noted on osteosarcoma cell proliferation and migration [31, 32], indicating that strict control of the amount of FOXC1 in the developmental process is essential to maintain cellular homeostasis. Recently, we reported that endogenous ROS production in the early stage of differentiation controls early events in spontaneous carcinogenesis [10]. By subcutaneous xenograft transplantation with ChiPSC12-M cells overproducing ROS in the initial stage of differentiation, we observed the formation of tumors in the backs of nude mice, suggesting that cells overproducing ROS have tumor-initiating abilities in the early stage of development. Impaired differentiation is closely associated with oncogenesis. Although some cancer stem cells have differentiation abilities, such abilities are limited in comparison with those of normal tissue stem cells [33–35]. Thereby, improvement of the impaired differentiation in cancer stem cells may be an effective treatment against cancer. It is known that *FOXC1* expression is negatively controlled by a class of small non-coding RNA molecules, microRNA-133b [16], and that oxidative stress inhibits the expression of tumor-suppressor microRNAs, including microRNA-133b [36]. Taken together, *FOXC1* is a candidate gene for the initial factor leading to carcinogenesis induced by internal ROS production. However, further study is needed to explore the mechanism of how transiently increased FOXC1 expression in the initial stage of differentiation induces impaired differentiation, resulting in carcinogenesis.

In conclusion, the present study highlights a novel approach to clarify the molecular pathways leading to oxidative stress-induced diseases including cancer.

## Methods

### Human induced pluripotent stem (iPS) cells

Cellartis Human iPS Cell Line 12 (ChiPSC12, Y00285) was purchased from Takara Bio Inc. (Shiga, Japan). The cell line was created by reprogramming human skin fibroblasts using defective polycistronic retrovirus technology to deliver c-Myc, Oct-4, SOX2, and KLF4 [19–22]. The cell line origin has been confirmed by authentication using banded chromosome analysis. The last analysis was carried out a month before the start of the experiments. *Mycoplasma* testing was performed and all cell lines were used within 3 months of their acquisition. The cells were cultured using the Cellartis DEF-CS Culture System (Y30010, Takara Bio Inc.) including Matrigel-based culture system, according to the manufacturer’s instructions at 37 °C in 5% CO_2_. The cells were differentiated into the definitive endoderm using the Cellartis Definitive Endoderm Differentiation Kit (Y30030, Takara Bio Inc.).

### Construction of plasmids to overexpress human SDHC (I69E) or mitochondrial-targeted human catalase, under the Tet-On ProteoTuner system

We used the pTRE-Cycle1 vector (631115, Clontech) to control endogenous ROS production under the Tet-On ProteoTuner system. Human SDHC cDNA cloning was performed as follows: wild-type SDHC PCR product was obtained from the cDNA template of ChiPSC12 cells by PCR using the following primer set (Forward, 5′-CGTCACTTCCGTCCAGA-3′; Reverse, 5′-ACAGAGCTGGCATTGTTTCCAC-3′). The mutant SDHC allele was amplified by 1st and 2nd PCR using the following primer sets (Forward, 5′-TTAAGTATACGCCACCATGGCTGCGCTGTTGCTG-3′; Reverse, 5′-GGCATTCGGACATCGCCATGGGAA-3′), (Forward, 5′-CCCACATTACTATCTACAGTTGGTC-3′; Reverse, 5′-AAAAAGATCTTCACATGGCTGCCAGCCC-3′). The first PCR product was amplified using the following primer set (Forward, 5′-TTAAGTATACGCCACCATGGCTGCGCTGTTGCTG-3′; Reverse, 5′-AAAAAGATCTTCACATGGCTGCCAGCCC-3′) and inserted into a pTRE-Cycle1 vector at the *Nde* I-*Xba* I site (Cycle1-SDHC (I69E) vector). The mutated nucleotide (ATC to GAA) in SDHC cDNA was confirmed by DNA sequencing. To construct the pTRE-Cycle1 vector that encodes both SDHC (I69E) and mitochondrial-targeted human catalase, wild-type catalase cDNA without the carboxy-terminal peroxisomal localization signal was obtained using the following primer set (Forward, 5′-ACTCGGGGCAACAGGCAGATTT-3′; Reverse, 5′-AGCCACTAGCTTGCATTTGCAC-3′). To stabilize catalase in mitochondria under the Tet-On ProteoTuner system, mitochondrial targeting sequence (MTS)-fused human catalase should be expressed as DD-tagged protein. However, N-terminal mitochondrial localization signal fused proteins dominantly translocate to mitochondria before recognition by Shield-1 protein, resulting in degradation [37]. Therefore, we constructed C-terminal MTS-fused human catalase using GSTA4-4 of mouse glutathione S-transferases (20 C-terminal amino acid residues, 172–222) [38–40]. GSTA4-4 was constructed by annealing four oligonucleotides sets and then ligated after phosphorylation.

F_ApaL_GSTA1/R_ApaL_GSTA1 (5′-TGCACGCCCCTGTACTGTCCGACTTCCCTCTGCTGCAGGCATTTAAGACAAGAATCAGCAACATTCCTACAATTAAGAAGTTCC-3′; 5′-GGAACTTCTTAATTGTAGGAATGTTGCTGATTCTTGTCTTAAATGCCTGCAGCAGAGGGAAGTCGGACAGTACAGGGGCG-3′). F_ApaL_GSTA1/R_ApaL_GSTA1 (5′-TGCACGCCCCTGTACTGTCCGACTTCCCTCTGCTGCAGGCATTTAAGACAAGAATCAGCAACATTCCTACAATTAAGAAGTTCC-3′; 5′-GGAACTTCTTAATTGTAGGAATGTTGCTGATTCTTGTCTTAAATGCCTGCAGCAGAGGGAAGTCGGACAGTACAGGGGCG-3′). F_GSTA5′2Hind/R_GSTA2Hind (5′-TGCAACCCGGAAGTCAGAGGAAGCCTCCTCCAGATGGCCCCTATGTTGAGGTGGTCAGGACTGTCCTGAAGTTCTAGTGA-3′; 5′-AGCTTCACTAGAACTTCAGGACAGTCCTGACCACCTCAACATAGGGGCCATCTGGAGGAGGCTTCCTCTGACTTCCGGGTTGCA-3′). To construct C-terminal MTS-fused catalase, catalase cDNA was amplified by the following primer sets (Forward, 5′-TTTTACGCGTGCTGACAGCCGGGAT-3′; Reverse, 5′-TTAAGTGCACAGATCCGGACTGCACAAAGG-3′). Catalase cDNA was ligated with GSTA4-4 following *Mlu* I/*ApaL* I digestion and finally inserted into the Cycle1-SDHC (I69E) vector at the *Mlu* I/*Hind* III site (Cycle1-SDHC (I69E)/DD-tagged mt-catalase vector). The cells were transfected with the Cycle1-SDHC (I69E)/DD-tagged mt-catalase vector using X fect ^TM^ (631317, Clontech). Stable transfectants were selected in DEF-CS culture medium (Takara Bio Inc.) containing 0.2 μg/mL puromycin and maintained in the presence of 0.1 μg/mL puromycin.

### Measurement of cell area on differentiation Day 7

To quantify the cell area, the dishes were observed under WRAYCAM NF500 (WRAYMER Inc., Osaka, Japan) equipped with Nikon TMS Inverted Microscope (MFA 10100, Nikon Corporation, Tokyo, Japan) and each cell area was measured using ImageJ 1.49v (NIH, MD, USA).

### Real-time quantitative RT-PCR analysis

Total RNA was prepared with an RNeasy mini kit (Qiagen) and used to synthesize cDNA by Transcriptor First Strand cDNA Synthesis Kit (Roche). SYBR Premix EX Taq II (Takara Bio Inc.) was used for real-time PCR with the CX96 Real Time PCR System (Bio-Rad). The primers used for PCR were as follows: SOX17-specific primers (Forward, 5′-ACTGCAACTATCCTGACGTG-3′; Reverse, 5′-AGGAAATGGAGGAAGCTGTT-3′), FOXC1-specific primers (Forward, 5′-TAAGCCCATGAATCAGCCG-3′; Reverse, 5′-GCCGCACAGTCCCATCTCT-3′), OCT3/4-specific primers (Forward, 5′-GACAGGGGGAGGGGAGGAGCTAGG-3′; Reverse, 5′-CTTCCCTCCAACCAGTTGCCCCAAAC), β-Actin-specific primers (Forward, 5′-TGGCACCCAGCACAATGAA-3′; Reverse, 5′-CTAAGTCATAGTCCGCCTAGAAGCA-3′), purchased from Takara Bio Inc. The expression levels of SOX17, FOXC1, and OCT3/4 were normalized by the β-actin expression levels.

### Immunostaining

Antibodies against catalase (1:200, ab16731, Abcam plc., Tokyo, Japan) and CXCR4 (1:50, sc-53534, Santa Cruz Biotechnology Inc.) were used. MitoTracker^TM^ red CXMRos as a mitochondrial marker was obtained from Thermofisher Scientific Inc. (MA, USA). To detect the expression of human catalase in mitochondria, ChiPSC12-CAT cells were cultured with medium containing Tet-Express solution for 24 h and treated with 0.8 μL Shield-1 solution for 3.5 h and then incubated with 1 μM MitoTracker for 20 min at 37 °C. The fixed cells were subjected to immunostaining using anti-catalase antibody. Digital images were separately captured from identical fields using an LSM-710 Meta confocal microscopy system (Carl Zeiss, Jena, Germany). Images were processed for publication by Adobe Photoshop CS5 software (Adobe System Inc., San Jose, CA, USA).

### Measurement of cell area and anti-catalase immunoreactivity

The cell area (the area occupied by a single cell) was detected by differential interference contrast (DIC) imaging and measured using ImageJ 1.49v. Images of anti-catalase immunoreactivity were converted to 256-level gray-scale images by Adobe Photoshop CS5 software and the signal intensities for each cell were measured using ImageJ 1.49 v. At least 50 cells were analyzed, and the integrated pixel density of each cell was calculated and is shown as the catalase index.

### Quantitative immunodetection of mitochondrial 8-oxoG

To solely detect 8-oxoG in mitochondrial DNA, slides were pretreated with RNase (5 mg/mL; Sigma-Aldrich) and were subjected to immunostaining with the anti-8-oxo-dG antibody (sc66036, 1:100, Santa Cruz). The image of 8-oxoG immunoreactivity was converted to 256-level gray-scale images and the signal intensities were measured in each digital image using ImageJ 1.49v. Mitochondrial 8-oxoG index was detected by subtracting the nuclear 8-oxoG immunofluorescence from the immunofluorescence of the whole cell [41]. Fluorescence intensities of mitochondrial 8-oxoG in the five digital images of each cell line are measured and the relative 8-oxoG index per cell is shown as a dot plot.

### siRNA and transfection

siRNA against human FOXC1 (SC-43766) was purchased from Santa Cruz Biotechnology Inc. Cells were transfected with *FOXC1*-siRNAs using Repofectamine 3000 (Invitrogen) on Day 0 before transferring to the differentiation medium.

### Statistical analysis and data presentation

All statistical analyses were carried out using JMP 8.01 software (SAS Institute, NC, USA).

No sample size determination was performed during the design of the experiments. For comparison between groups, one-way ANOVA with the Tukey–Kramer HSD test was used. Normal distribution and homoscedasticity of data were confirmed by the Shapiro–Wilk test and Levene’s tests, respectively. Data that were not normally distributed were analyzed by the Wilcoxon exact test (two-sided). The experiments were carried out using a highly standardized protocol, and blind analysis was used for all data.

## Data Availability

All data generated or analysed during this study are included in this published article.
